# Changes in sensory, postural stability and gait functions depending on cognitive decline, and possible markers for detection of cognitive status

**DOI:** 10.1186/s12911-022-01955-x

**Published:** 2022-09-22

**Authors:** Emilija Kostic, Kiyoung Kwak, Dongwook Kim

**Affiliations:** 1 Department of Healthcare Engineering, The Graduate School, Jeonbuk National University, 567 Baekje-daero, Deokjin-gu, Jeonju-si, Jeollabuk-do Republic of Korea; 2 Division of Biomedical Engineering, College of Engineering, Jeonbuk National University, 567 Baekje-daero, Deokjin-gu, Jeonju-si, Jeollabuk-do Republic of Korea; 3 Research Center for Healthcare & Welfare Instrument for the Elderly, Jeonbuk National University, 567 Baekje-daero, Deokjin-gu, Jeonju-si, Jeollabuk-do Republic of Korea

**Keywords:** Mild cognitive impairment (MCI), Gait, Sensory systems, Older adults, Biomarkers, Dementia

## Abstract

**Background:**

Numerous people never receive a formal dementia diagnosis. This issue can be addressed by early detection systems that utilize alternative forms of classification, such as gait, balance, and sensory function parameters. In the present study, said functions were compared between older adults with healthy cognition, older adults with low executive function, and older adults with cognitive impairment, to determine which parameters can be used to distinguish these groups.

**Results:**

A group of cognitively healthy older men was found to have a significantly greater gait cadence than both the low executive function group (113.1 ± 6.8 vs. 108.0 ± 6.3 steps/min, *p* = 0.032) and the cognitively impaired group (113.1 ± 6.8 vs. 107.1 ± 7.4 steps/min, *p* = 0.009). The group with low executive function was found to have more gait stability than the impaired cognition group, represented by the single limb support phase (39.7 ± 1.2 vs. 38.6 ± 1.3%, *p* = 0.027). Additionally, the healthy cognition group had significantly greater overall postural stability than the impaired cognition group (0.6 ± 0.1 vs. 1.1 ± 0.1, *p* = 0.003), and the low executive function group had significantly greater mediolateral postural stability than the impaired cognition group (0.2 ± 0.1 vs. 0.6 ± 0.6, *p* = 0.012). The low executive function group had fewer mistakes on the sentence recognition test than the cognitively impaired (2.2 ± 3.6 vs. 5.9 ± 6.4, *p* = 0.005). There were no significant differences in visual capacity, however, the low executive function group displayed an overall greatest ability.

**Conclusions:**

Older adults with low executive function showcased a lower walking pace, but their postural stability and sensory functions did not differ from those of the older adults with healthy cognition. The variables concluded as good cognitive status markers were (1) gait cadence for dividing cognitively healthy from the rest and (2) single limb support portion, mediolateral stability index, and the number of mistakes on the sentence recognition test for discerning between the low executive function and cognitive impairment groups.

## Background

Cognitive deterioration is a worldwide issue affecting both individual and societal psychological and economic well-being. According to Alzheimer’s Disease International, the number of people affected by dementia is expected to increase to 82 million by 2030 and reach 152 million by 2050 due to the aging of the world population [1]. However, this number may be even greater than reported. A recent study reported that 91.4% of older individuals with a cognitive impairment consistent with dementia received no diagnosis relating to dementia [2]. This percentage can be assumed to be even higher for those with mild cognitive impairment (MCI), which has the potential to progress to dementia. This absence of diagnosis leads to the absence of treatment until the disease progresses to severe dementia. A survey carried out by Alzheimer’s Society has found that 62% of people felt a dementia diagnosis would mean their life was over, making fear of diagnosis the number one reason for not seeking out professional help [3]. Such a serious issue can be addressed by actualizing systems that assess the cognitive status without the need for traditional cognitive assessments.

In recent years attention has been given to motor and sensory impairments as prodromal markers of cognitive decline. For instance, slowing of the gait, shorter stride length, and relying more on the double limb stance during walking were linked to a higher risk of cognitive impairment [4–7]. According to Albers [8], the auditory, visual, and vestibular systems, as well as the areas of the central nervous system that are responsible for sensory and motor functions, are impaired by Alzheimer’s dementia pathology. Through various studies, it was discovered that auditory [9–11] and visual [10–12] impairments have a relation to incidence of dementia. The aforementioned research has mostly targeted the differences between cognitively healthy and dementia patients and observed significant differences. However, in order to enable early detection and cognitive maintenance or rehabilitation, differences and similarities in gait, balance, and sensory functions of older adults in several stages of the disease progression need to be assessed.

Therefore, the aim of the present study was (1) to compare gait, postural stability, the auditory and visual ability of healthy older adults, older adults with low executive function, and older adults with cognitive impairment and (2) to assess the discriminative capacity of the aforementioned functions’ parameters in determining the cognitive status.

## Results

### Demographics

Out of the 72 participants, 19 had MoCA scores compatible with the presence of cognitive impairment and were classified as impaired cognition (IC), and among the remaining 53, 19 who failed to complete the TMT-KL test were classified as low executive function (LEF), and 34 were classified as healthy cognition (HC). Among the IC, no participant completed the TMT-KL. The demographics of the 3 groups are presented in Table 1. There were no statistically significant differences observed.

**Table 1:** Descriptive characteristics of the participants

Variable	HC (n = 34)	LEF (n = 19)	IC (n = 19)
	Mean (SD)	Mean (SD)	Mean (SD)
Age (years)	73.6 (4.6)	73.9 (3.9)	76.3 (4.0)
Height (cm)	168.8 (5.6)	165.0 (6.0)	166.2 (4.9)
Weight (kg)	66.2 (9.4)	66.4 (6.8)	66.9 (7.9)
Years of education	13.3 (2.8)	13.8 (4.0)	13.0 (3.5)

*HC* healthy cognition, *LEF* low executive function, *IC* impaired cognition, *SD* standard deviation

### Cognitive testing

**Fig. 1:**
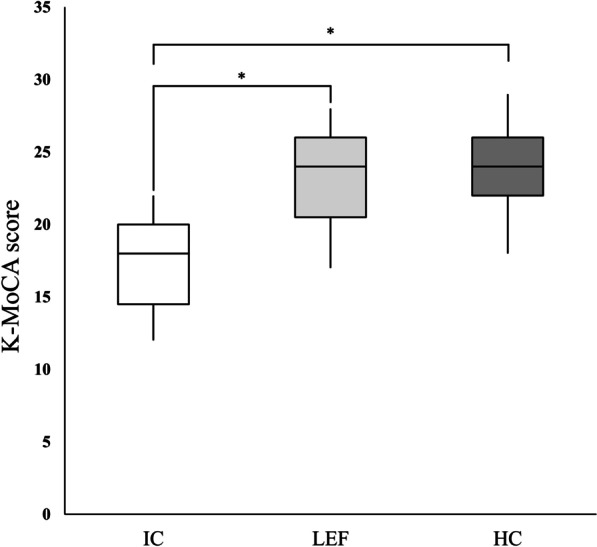
Montreal cognitive assessment (MoCA) score distributions for impaired cognition (IC) group, low executive function (LEF) group, and healthy cognition (HC) group; *p < 0.05

In terms of cognitive ability, a significant difference (*p* < 0.001) was observed, and post hoc analysis revealed that HC and LEF groups did not differ between each other but were significantly greater than IC, both with a *p* value of < 0.001 (Fig. 1).

### Level walking

The level walking variables revealed significant differences between the three groups. The groupwise average values and the corresponding *p* values can be seen in Table 2.

**Table 2:** Gait function parameters of the healthy cognition group, low executive function group and impaired cognition group

Variable	HC (n = 34)	LEF (n = 19)	IC (n = 19)	*p* value	Post-hoc		
	MEAN (SD)	MEAN (SD)	MEAN (SD)		HC-LEF	HC-IC	LEF-IC
Step length (cm)	66.4 (6.6)	64.2 (5.2)	63.2 (5.2)	NS	–	–	–
Stride length (cm)	128.4 (10.8)	124.5 (9.2)	123.0 (9.1)	NS	–	–	–
Gait velocity (cm/sec)	121.2 (14.0)	112.3 (13.0)	110.2 (13.9)	0.011	NS	0.021	NS
Cadence (steps/min)	113.1 (6.8)	108.0 (6.3)	107.1 (7.4)	0.004	0.032	0.009	NS
Stride (ms)	1065.6 (64.3)	1115.4 (65.5)	1127.4 (84.1)	0.004	0.016	0.013	NS
Stance (ms)	648.2 (47.5)	689.0 (50.8)	696.8 (59.9)	0.003	0.020	0.018	NS
Swing (ms)	417.4 (21.3)	428.1 (18.5)	430.5 (28.9)	NS	–	–	–
SLSP (%)	39.5 (1.2)	39.7 (1.2)	38.6 (1.3)	0.019	NS	NS	0.027
DLSP (%)	21.3 (1.9)	21.9 (1.8)	23.1 (2.3)	0.008	NS	0.006	NS
SPP (%)	39.2 (1.1)	38.3 (1.2)	38.2 (1.2)	0.003	0.022	0.009	NS
LR (%)	10.4 (1.1)	10.7 (1.2)	11.4 (1.5)	0.016	NS	0.013	NS
MS (%)	21.2 (1.9)	20.8 (3.1)	19.6 (2.4)	NS	–	–	–
TS (%)	18.3 (2.3)	18.9 (3.4)	19.0 (2.4)	NS	–	–	–
PS (%)	10.9 (1.1)	11.2 (0.8)	11.7 (1.1)	0.024	NS	0.020	NS

*HC* healthy cognition, *LEF* low executive function, *IC* impaired cognition, *SLSP* single limb support portion, *DLSP* double limb support portion, *SPP* swing phase portion, *LR* loading response, *MS* mid-stance, *TS* terminal stance, *PS* pre-swing, *NS* no significance, *SD* standard deviation

Significance level *p* < 0.05

In the case of cadence, stride and stance duration, and swing phase portion, the HC group was found to have significantly different results than both LEF and IC. In the case of velocity, loading response phase, pre swing phase, and the double limb support portion, there were significant differences only between HC and IC. For all these variables, a trend can be observed where the LEF group average value lies between the average values of HC and IC.

The only variable where a significant difference between LEF and IC occurred is the single limb support portion, and in this case, the LEF group displayed the highest average value. Among the variables representing the gait function, to distinguish HC from LEF as well as HC from IC, cadence can be used for its high AUC (CI 95%) of 0.729 (0.586–0.872) and 0.728 (0.588–0.867), respectively. When discerning between LEF and IC, SLSP had the highest AUC (CI 95%) of 0.727 (0.566–0.889).

### Postural stability

All the postural stability indexes revealed significant differences between the groups. These differences and the corresponding *p* values are presented in Table 3.

**Table 3:** Postural stability indexes of the healthy cognition group, low executive function group and impaired cognition group

Variable	HC (n = 34)	LEF (n = 19)	IC (n = 19)	*p* value	Post-hoc		
	MEAN (SD)	MEAN (SD)	MEAN (SD)		HC-LEF	HC-IC	LEF-IC
PSI	0.6 (0.1)	0.7 (0.4)	1.1 (0.8)	0.003	NS	0.003	0.027
APSI	0.4 (0.1)	0.6 (0.4)	0.8 (0.6)	0.013	NS	0.011	NS
MLSI	0.2 (0.1)	0.2 (0.1)	0.6 (0.6)	0.008	NS	0.033	0.012

*HC* healthy cognition, *LEF* low executive function, *IC* impaired cognition, *PSI* postural stability index, *APSI* AnteroPosterior Stability Index, *MLSI* MedioLateral Stability Index, *NS* no significance, *SD* standard deviation

Significance level *p* < 0.05

In the case of anteroposterior stability index, a significant difference was observed only between HC and IC, while PSI and MLSI displayed significant differences between HC and IC as well as LEF and IC. The overall amount of swaying was the lowest in HC, followed by LEF and finally IC. When discerning between HC and IC the PSI variable displayed the highest AUC (CI 95%) of 0.784 (0.651–0.917), and when discerning between LEF and IC it was MLSI with AUC (CI 95%) = 0.763 (0.612–0.914).

### Audiological exam

Table 4 presents the audiological variables’ average values and the corresponding *p* values.

**Table 4:** Audiological parameters of the healthy cognition group, low executive function group and impaired cognition group

Variable	HC (n = 34)	LEF (n = 19)	IC (n = 19)	*p* value	Post-hoc		
	MEAN (SD)	MEAN (SD)	MEAN (SD)		HC-LEF	HC-IC	LEF-IC
PTA512	23.0 (11.3)	21.4 (9.4)	24.0 (10.1)	NS	–	–	–
WRS	73.8 (15.9)	79.1 (10.3)	72.6 (12.8)	NS	–	–	–
WRS error	12.5 (7.2)	10.5 (5.1)	13.8 (6.5)	NS	–	–	–
SRS	97.3 (2.9)	98.5 (2.1)	95.2 (4.7)	0.008	NS	NS	0.006
SRS error	3.3 (4.3)	2.2 (3.6)	5.9 (6.4)	0.006	NS	NS	0.005

*HC* healthy cognition, *LEF* low executive function, *IC* impaired cognition, *SRS* sentence recognition score, *NS* no significance, *SD* standard deviation

Significance level *p* < 0.05

In sentence recognition testing, the LEF group obtained the highest score on average, with the lowest number of mistakes. The average number of mistakes made by LEF was more than two times lower than that of IC resulting in a significant difference between these two groups. The remaining variables revealed no statistically significant differences, however the LEF group had the greatest auditory capacity on average. SRS error variable can be utilized to discern LEF from IC with an AUC (CI 95%) of 0.787 (0.643–0.930). In addition, Spearman bivariate analysis was performed to examine the correlation of PTA512 with the other variables. No significant correlation was found between PTA512 and SRS or SRS error, but there was significant correlation between PTA512 and both WRS and WRS error. Therefore, WRS and WRS error were adjusted for the effect of PTA512 and compared once more between the groups. This analysis found no significance between the groups in regard to WRS and WRS error.

### Ophthalmological exam

**Fig. 2:**
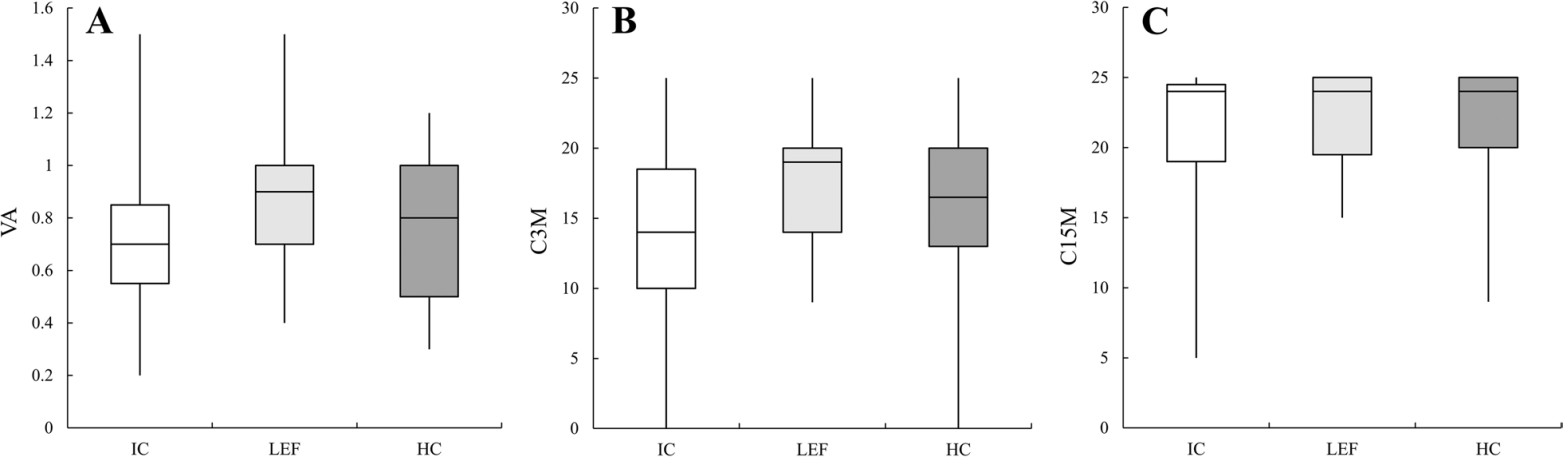
The visual ability of the impaired cognition (IC) group, low executive function (LEF) group, and healthy cognition group (HC): **A** visual acuity, **B** contrast sensitivity score at 3 m distance and **C** contrast sensitivity score at 1.5 m distance

The ophthalmological examination revealed no significant differences between the three groups. However, the LEF group displayed the highest average ability in the case of best-corrected visual acuity (Fig. 2A), and contrast sensitivity score at 3 m and 1.5 m distances (Fig. 2B, C).

The post-hoc power analysis for α = 0.05 and an effect size of 0.4, which is considered a large effect size in the case of ANOVA [13], resulted in power of approximately 0.85.

### Multiple sensory, gait, and balance abilities

**Fig. 3:**
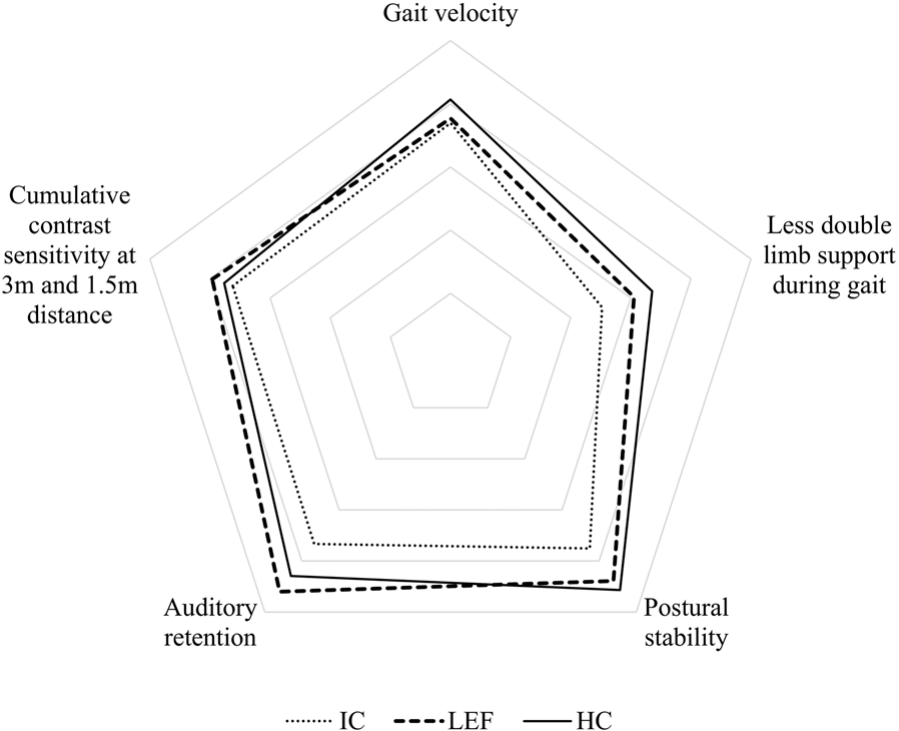
Comparison of multiple sensory and gait ability between older adults with healthy cognition (HC), older adults with low executive function (LEF) and cognitively impaired older adults (IC)

To visualize the abilities of each group, a radial graph with five axes was utilized (Fig. 3). These axes represent gait velocity, less relying on double limb support, balance, sentence retention, and cumulative contrast sensitivity score. The double limb support, postural stability, and sentence retention are represented by variables where a lower number indicates greater performance and were therefore inverted for visualization. All the variables were scaled to 100% using the respective maximum value in the given sample. The IC group has shown a low ability in every segment. On the other hand, the HC group shows a greater gait and balance performance, but the auditory and visual ability is greater in the LEF group.

## Discussion

When the cognitive function of the 3 groups was examined, the LEF group displayed a similar MoCA score average as the HC group, despite the lower executive function. Both groups were found to have a significantly higher score than the IC group, showing that the cognitive test score of the LEF group is not a good indicator of executive function deterioration. The demographic variables showed no difference between the three groups, indicating that an adjustment for covariates is not needed when comparing these groups’ functions.

When analyzing the gait variables, LEF and IC had mostly similar performance, with the exclusion of the single limb support portion where there was a significant difference between these two groups. The gait ability of HC was found to be significantly greater than that of IC in the case of velocity, cadence, stride duration, stance duration, loading response phase, double limb support phase, and swing phase. Among these variables cadence, stride duration, stance duration, and swing phase portion displayed a significant difference between HC and LEF. These results show that the LEF group has a significantly slower walking pace than HC, similar to that of the IC group, indicating that this group can be considered at risk of cognitive impairment because a slowing of the gait was determined a predecessor to the cognitive deterioration [4, 6, 14]. On the other hand, longer loading response and double limb support phase, as well as the shorter single limb support phase present in IC, are all indicators of poor stability during gait, which is not the case with LEF.

During LR the bodyweight needs to be transferred onto a limb that just completed its forward swing [15]. To enable this, the vestibular system needs to accurately determine which limb position would result in retaining balance. It has been suggested that there is a phase-based vestibular information weighing, and according to Bent [16], vestibular information received at heel contact is held at higher importance when walking. One hypothesis is that a long time of gait cycle spent in LR indicates a slower adjustment of the vestibular system. Such would imply that the IC participants have an impairment of the vestibular system. The LR and PS phases are sub-phases of double limb support, so the increased percentage spent in LR, and PS is accompanied by the increase in double limb support percentage. On the other hand, the single limb support percentage can be regarded as a representative index of support capacity of the affected limb [15]. Therefore, a shorter percentage would indicate less support capacity, or in other words, less stability.

According to the gait function analysis results, participants in the IC group exhibit poorer balance than those in the HC and LEF groups. Postural stability testing confirmed this indication. There were significant differences between HC and IC for all postural stability indexes and there were significant differences between LEF and IC in the case of mediolateral and overall postural stability index. As suggested by earlier studies, this finding brings further confirmation that low postural stability is a sign of cognitive deterioration [17].

The results of pure tone audiometry revealed the highest average ability in LEF. However, apart from the sentence recognition test score and number of mistakes, the auditory variables showed no significant differences between the three groups. The LEF group was shown to have the greatest auditory retention ability. This ability is closely related to attention and perceptual processing, as is visual capacity. Similar to the auditory ability, even though the statistical analysis showed no significant differences between the groups, the LEF group was found to have the greatest visual capacity.

Based on the post-hoc power analysis it can be concluded that the statistical tests that were performed had enough power to detect large effect sizes. Therefore, the aforementioned variables presented large statistically significant differences between the respective groups.

Sensory, postural stability and gait functions of the three groups have shown that some variables have a high discriminative ability. The variables that were found to be good at discerning between groups and could therefore be used in detection systems were (1) cadence during level walking for dividing cognitively healthy older individuals from the rest and (2) single limb support portion, mediolateral stability index, and the number of mistakes on the sentence recognition test for discerning between older adults at risk of cognitive impairment from the ones with cognitive impairment.

The radial graph representing gait, balance, and sensory functions shows that the sensory ability is on average the highest in the LEF group, despite the gait and postural ability being the highest in the case of HC. Therefore, the question is: what does this say about the sensory and cognitive function association? Several hypotheses attempt to explain how the sensory systems and cognitive function are associated, one of which is the information degradation hypothesis. According to this hypothesis, when the sensory periphery is impaired, the degraded sensory input places an increased demand on the processing resources. These resources are considered to be limited in the amount of information that can be held in memory [18]. For example, when the quality of the auditory signal is degraded by environmental noise, or hearing loss, the ‘listening effort’ needed for processing and comprehending increases. This in turn diverts the limited cognitive resources towards effortful listening [19, 20], leaving no cognitive resources available for other tasks. It has also been suggested that the age-related cognitive changes stem from the age-related changes in sensory processing [21]. In a study done by Karawani [22], two groups of hearing-matched older adults, one that was given a hearing aid for the first time and one without it, were compared after a period of 6 months. At the end of the trial, enhanced working memory performance and increased cortical response were observed in the group with a hearing aid. These findings suggest that sensory restoration can free up available cognitive resources for remembering the spoken conversation. As our results showed, the number of mistakes participants made while recalling sentences was not correlated to the pure tone audiometry results. This could be due to sentence retention requiring more cognitive resources than word retention or listening for a pure tone. Hence, the effort put into listening may be enough for the cognitively impaired to hear the tones or words played for them just as well as the ones with normal cognition, but not enough to accurately remember whole sentences. In regard to vision, untreated poor vision was found to be a contributing factor to dementia in older individuals [23], and patients with dementia used less visual correction, had fewer ophthalmological treatments, and underwent fewer ocular surgeries [24]. Such findings indicate that treating the sensory periphery can aid in stopping or even reversing cognitive decline.

### Limitations

One limitation of this study is the absence of longitudinal data which would follow the course of cognitive function of the participants. However, because in longitudinal studies a percentage of participants drops out, a larger sample is needed to reach meaningful conclusions. Therefore, another limitation is the size of the dataset, as a larger sample size would allow for more precise distinction between groups and even in designing a classification model. Additionally, higher resolution testing may reveal more about the visual ability of the three groups.

## Conclusions

A significantly large percentage of older adults that present with a cognitive impairment consistent with dementia never receive a formal medical diagnosis of the condition. This percentage can be assumed to be even higher for people with mild cognitive impairment, which has the potential to progress to dementia. The absence of diagnosis leads to the absence of treatment or possible prevention. Such a serious issue can be addressed by forming systems of detection of cognitive status and determining methods for maintaining or rehabilitating the cognitive function.

The present study assessed the differences in gait, balance, and sensory functions of cognitively healthy older individuals, those who scored above the cutoff points on the cognitive test but have lower executive function, and older adults with cognitive impairment. Participants were divided into 3 groups based on their K-MoCA score and executive function score. The statistical analysis showed that lower executive function coincides with slower walking pace, similar to that of the cognitively impaired. However, despite the slowing of the gait, the group with lower executive function showed greater balance, similar to that of the cognitively healthy. Additionally, this group showed the best average auditory and visual capacity among the 3 groups, with significantly higher auditory retention than the cognitively impaired. It was determined that cognitively healthy older individuals could be discerned from the rest by using the gait cadence variable, due to its high AUC. For discerning older adults with lower executive function from the ones with cognitive impairment, single limb support portion, mediolateral stability index and the number of mistakes on the sentence recognition test can be used as markers.

By utilizing the findings from the present study, detection systems that determine the cognitive status can be actualized to decide whether a patient has healthy cognition, lower executive function or impaired cognition. This would aid clinical practice by allowing clinicians to detect signs of early cognitive deterioration during regular check-ups.

## Methods

### Participants

The present study examined cognitive, gait, audiological, ophthalmological, and postural stability functions of 72 healthy community-dwelling Korean men older than 65. The participants completed two sessions of experiments executed on two different days. During the first session, demographic, cognitive, and gait function data were obtained. The demographic variables are as follows: age, height, weight, and years of education. Height and weight were measured during the first visit, age was confirmed from the participants’ identification document and years of education were self-reported. During the second session, audiological, ophthalmological, and postural stability functions were measured. All participants provided written informed consent prior to participation. The study received approval from the Jeonbuk national university institutional review board (JBNU IRB File No. 2019-09-015-001).

### Cognitive testing

Cognitive performance of the participants was assessed using the Korean version of the Montreal cognitive assessment (K-MoCA). Participants whose scores were compatible with the presence of cognitive impairment were placed in the impaired cognition (IC) group, using the cutoff points from a normative study [25]. The given cutoff points differ based on age and education and range from 6 to 26 points. The K-MoCA test examines seven cognitive abilities: visuospatial executive function; naming; attention; language; abstraction; delayed recall; and orientation. The first question of the visuospatial executive function section, a modified trail making test with Korean letters (TMT-KL), was used to divide the participants who scored above their respective cutoff points into participants with lower executive function (LEF) and ones with healthy cognition (HC). This test is equivalent to the trail-making test B (TMT-B), except it consists of only 5 numbers and 5 letters, and instead of the letters of the English alphabet, Korean letters are used. In the K-MoCA test, the TMT-KL score is a categorical variable describing whether the participant completed the test without mistakes. The trail making test is useful in evaluating mental flexibility because of the required shifting between numbers and letters [26] and is a measure of executive function, specifically problem solving [27], which has been shown to be impaired in all types of mild cognitive impairment (MCI) [28]. Additionally, a cutoff of one mistake on the TMT-B was found to be a fairly good discriminator between cognitively healthy and cognitively impaired [29]. Participants in said study who had no mistakes on the TMT-B also had significantly higher MMSE scores, indicating a higher cognitive ability. For this reason, the participants whose scores indicate normal cognition but have not completed the TMT-KL can be considered as being at risk of progressing to mild cognitive impairment.

### Level walking

**Fig. 4:**
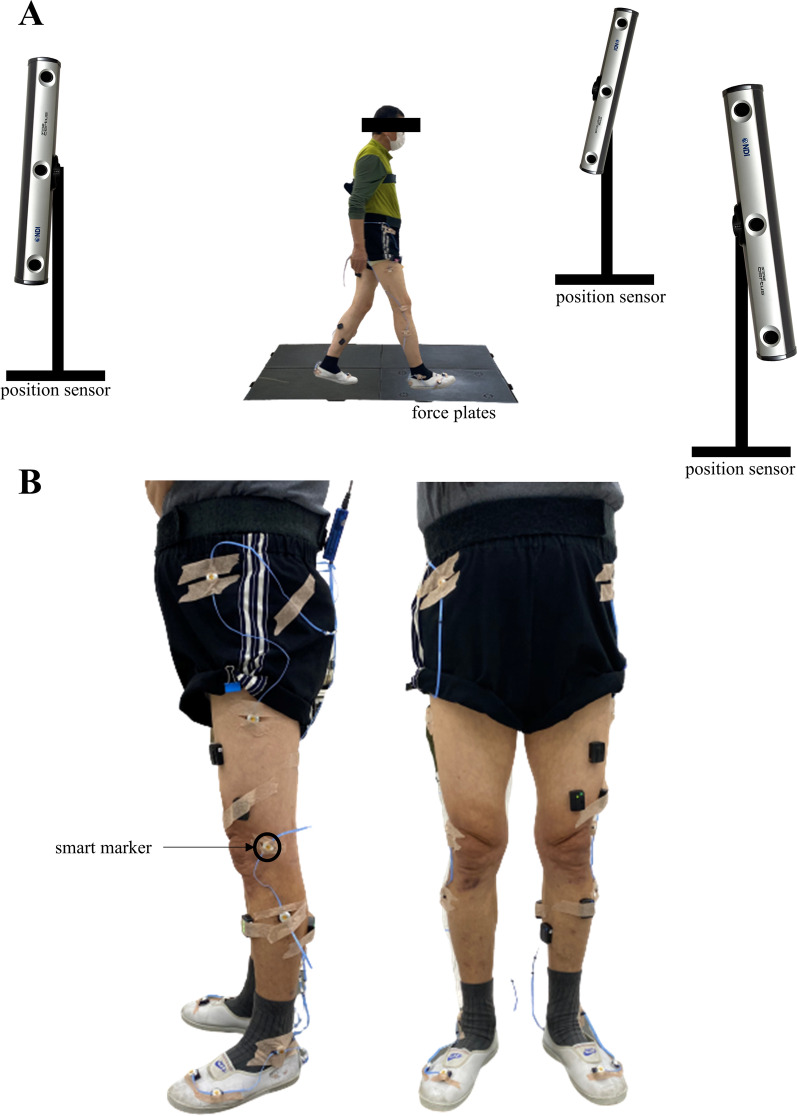
Motion capture and analysis system. **A** Position sensors and walkway, **B** lower limb marker placement: lateral and anterior view

Gait function measurements were obtained through a level walking task. A 10 m long walkway and software for capturing human motion (First Principle, Northern Digital Inc., Canada) were used to capture the participants’ gait alongside position sensors (Optotrak Certus, Northern Digital Inc., Canada) and force platforms (Bertec Ltd, USA). Motion module marker guide (MusculoGraphics, Inc., USA), which is the standard method for motion analysis [30], was referenced when placing infrared LED markers (Smart marker, Northern Digital Inc., Canada) on the participants’ legs, as shown in Fig. 4. Participants were asked to walk at their most comfortable pace. Software for Interactive Musculoskeletal Modeling (SIMM, Motion Analysis Corp., USA) was used to extract the gait variables for which an ensemble average of three trials was used as the final value. The variables extracted were the spatio-temporal variables, stance, stride, and swing duration and the subdivisions of the gait cycle. All variables were calculated in reference to the left foot heel contact.

### Postural stability testing

Postural stability measurements were performed using the Balance system SD (Biodex Medical System. Inc., USA). Participants completed the testing both feet firmly on the platform with their eyes open. The variables obtained were the overall postural stability index (PSI), anteroposterior stability index (APSI), and mediolateral stability index (MLSI) which are calculated as follows:

1 $$PSI=\sqrt{\frac{\sum {\left(0-x\right)}^{2}+\sum {\left(0-y\right)}^{2}}{n}}$$

2 $$APSI=\sqrt{\frac{\sum {\left(0-y\right)}^{2}}{n}}$$

3 $$MPSI=\sqrt{\frac{\sum {\left(0-x\right)}^{2}}{n}}$$

where n is the number of samples, and the indexes represent the amount of deviation from the point of origin. The balance system regards the center of the foot platform as the point of origin, making initial calibration a necessity. The participants must stand comfortably on the foot platform with their center of mass positioned above the point of origin before beginning the test.

### Audiological exam

The equipment used for auditory testing was a Korean speech audiometry test and an audiometer (GSI-61, Grason-Stadler, Denmark). The obtained parameters are as follows: (1) PTA512—a better ear average of the pure tone audiometry scores at 0.5, 1, and 2 kHz; (2) WRS—The word recognition score average; (3) WRS error—Total number of mistakes when recalling words; (4) SRS—The sentence recognition score average and (5) SRS error—Total number of mistakes when recalling sentences.

### Ophthalmological exam

Ophthalmological examination consisted of visual acuity and contrast sensitivity testing. Testing was performed with or without correction glasses, depending on the participants’ usual preference, to accurately assess their day-to-day ability.

Visual acuity was measured for both eyes using the Korean standard 3M vision chart. For contrast sensitivity, Lea Numbers 10M Flip chart (Lea test intl. LLC, Finland) was presented to the participants, first from a 3 m distance and then from a 1.5 m distance. The parameters obtained from the ophthalmological assessment and their respective explanations are as follows: (1) VA—The best-corrected visual acuity; (2) CS3M—Contrast sensitivity score at a 3 m distance and (3) CS15M—Contrast sensitivity score at a 1.5 m distance.

### Statistical analysis

Normality assessment of the variables was performed via the Shapiro–Wilk test. One-way ANOVA with Bonferroni post hoc analysis was performed for parametric data and the Kruskal–Wallis test with Dunn post hoc analysis was used for non-parametric data. For all the variables the area under the receiver operating characteristic curve (AUC) was assessed to determine their discriminative ability and whether they can be used as markers for detecting cognitive status.

All statistical analyses were executed using Statistical Package for the Social Sciences (SPSS) version 20.0.0 for Windows software (IBM Corp, USA). All average values are presented in the format MEAN (SD) and the significance level of all tests was defined as α = 0.05. Additionally, G*Power, a freely available software [31] was used to perform post-hoc power analysis.

## Data Availability

The datasets used and/or analyzed to reach the conclusions presented in this manuscript can be found at: https://www.synapse.org/#!Synapse:syn30365140 under the terms and conditions which can be found at the synapse repository.

## Abbreviations

MCI: Mild cognitive impairment
HC: Healthy cognition
IC: Impaired cognition
LEF: Low executive function
K-MoCA: Korean Montreal Cognitive Assessment
LR: Loading response
MS: Mid stance
TS: Terminal stance
PS: Pre-swing
SLS: Single limb support
DLS: Double limb support
SW: SWing phase
PTA: Pure tone audiometry
WRS: Word recognition score
SRS: Sentence recognition score
APSI: Anterior–posterior stability index
MLSI: Medio-lateral stability index
PSI: Postural stability index
SD: Standard deviation
SPSS: Statistical Package for the Social Sciences
ROC: Receiver operating characteristic
AUC: Area under the curve
TMT-KL: Trail making test with Korean letters
TMT-B: Trail making test B
CI: Confidence interval
S.E.: Standard error
